# An Early Diagnosis of an Ovarian Steroid Cell Tumor Not Otherwise Specified in a Woman

**DOI:** 10.1155/2019/2537480

**Published:** 2019-01-16

**Authors:** Patrícia Alves, Inês Sá, Miguel Brito, Cátia Carnide, Osvaldo Moutinho

**Affiliations:** Department of Gynecology and Obstetrics, Trás-os-Montes and Alto Douro Hospital Center, Avenida da Noruega, Lordelo, 5000-508 Vila Real, Portugal

## Abstract

Steroid cell tumor not otherwise specified is a subtype of steroid ovary tumors. These are a rare subgroup of ovarian sex cord-stromal tumors. We present a case of a young woman submitted to laparoscopic cystectomy with ovarian steroid cell tumor as histological finding. This represents the second case where laparoscopic cystectomy is performed in this type of tumor. Up to 36 months after surgery, the woman remains under surveillance, without recurrence of the tumor, and attempts to conceive. This case suggests that steroid cell tumors can be asymptomatic, and gynecologists must think about preserving fertility in women of reproductive age.

## 1. Introduction

Ovarian steroid cell tumors are a rare subgroup of sex cord-stromal tumors of the ovary and comprise less than 0,1% of all ovarian tumors. According to their cell of origin, they have been divided into three subtypes: stromal luteoma, Leydig cell tumor, and steroid cell tumor not otherwise specified (NOS) [1, 2].

In literature, only a few cases of steroid cell tumors NOS have been described, most with symptoms and bulky tumors at presentation. This report describes a case of an early diagnosis of steroid cell tumor NOS.

## 2. Case Presentation

A 30-year-old nulligravida presented herself in our institution because of the inability to conceive for four years. She was healthy, with no history of previous surgeries. Menarche had occurred at age 14. She had used oral contraceptives for nine years, having stopped taking them four years ago, followed by regular menses. No one in her family had history of infertility or any type of gynecologic cancer.

General examination and body mass index were normal. Analytically she had normal blood count and hormonal assay [FSH, LH, TSH, free T4, testosterone, and dehydroepiandrosterone (DHEA)]. A transvaginal ultrasonography was performed revealing a normed uterus and ovaries slightly enlarged and polycystic with a small echogenic mass in the right ovary of 15 millimeters. The hysterosalpingography was normal.

Her husband was healthy and had no relevant past medical history and his semen analysis was normal.

She was proposed for a diagnostic laparoscopy and ovarian drilling. In the surgery, both ovaries were polycystic and a yellowish nodular solid and hardened formation of about two centimeters, vascular and friable to touch, was observed in the right ovary. That mass was removed, without rupture, and removed in an endoscopic bag. Tubal patency test confirmed bilateral permeability.

**Figure 1:**
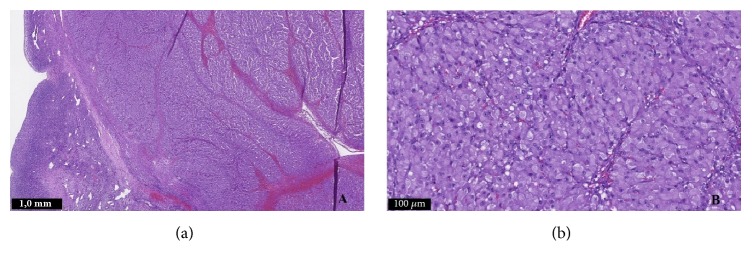
Hematoxylin and eosin staining of the surgical specimen.

**Figure 2:**
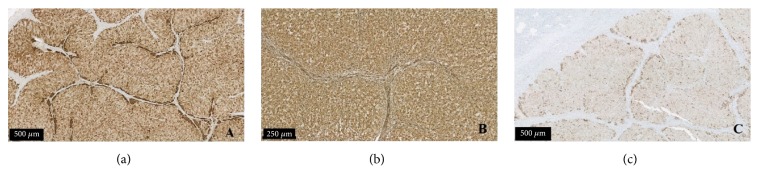
Immunohistochemical staining. Marking (a) for calretinin; (b) for vimentin; and (c) for melan-A.

The removed mass was sent to histological examination. Macroscopically, it was a yellow-colored tumor measuring two centimeters in greatest diameter, with a smooth and well-limited surface. Microscopically, the ovarian parenchyma was almost entirely occupied by a tumor lesion, represented by large polyhedral cells with small nuclei and vacuolated eosinophilic cytoplasm, without mitosis and nuclear atypia. Hemorrhage and necrotic areas were absent (Figure 1). Immunohistochemical study revealed diffuse marking for calretinin, vimentin, and melan-A (Figure 2). These features were consistent with ovarian steroid cell tumor NOS.

In the postoperative period, the woman is followed up with serial measurements of serum testosterone levels and transvaginal ultrasound. At 24 months, she had no evidence of recurrence and began follow-up in reproductive medicine consultation. She was proposed to* in vitro* fertilization treatment, but despite gonadotropin therapy (in high doses, 300U) for ovarian stimulation, she had no response. At 36 months postoperatively, she is still not pregnant and no evidence of recurrence too.

## 3. Discussion

Steroid cell tumor NOS is the most common of the ovarian steroid cell tumors, accounting for about 60%, and is thus called because the cell lineage is unknown [1, 2]. It can occur at any age, with 43 years being the mean age at diagnosis [3, 4]. This tumor can present as abdominal pain but the more significant clinical manifestations of this type of tumor are associated with the hormonal activity and virilizing properties, occurring in 56-77% of patients [1]. It may cause precocious puberty in children, and in adults it can manifest as oligomenorrhea, hirsutism, acne, increased libido, and deepening of the voice, for example [1, 3, 4]. It can also present estrogenic manifestations (6-23%), such as menorrhagia or postmenopausal bleeding. In 6-10% of cases, Cushing syndrome occurs and approximately in 25% of cases, such as this one, patients lack endocrine symptoms [5].

In virilized patients, elevated serum testosterone levels, more than 2,0 ng/mL, normal DHEA-S, and no evidence of 21 *α*-hydroxylase deficiency are strong indicators of an ovarian virilizing tumor or ovarian hyperthecosis [2, 4].

Most of these tumors are slow growing, diagnosed at an early stage, and symptoms are frequently present for many years before the diagnosis is made. They are unilateral in 94% of cases, large at diagnosis, and, typically, solid and well-circumscribed tumors [2, 4]. The color of the cut surface is often yellow but depends on the lipid content [3, 6]. Definitive diagnosis is made by histology. In addition to the microscopic features, immunohistochemistry is very helpful in diagnosing these tumors correctly. Calretinin and inhibin stain are found to be most useful in differentiating sex cord-stromal from non-sex cord-stromal tumors, because the first is positive for these two markers [1, 4]. Despite melan-A not being a marker described in the articles relating to ovarian steroid cell tumor, this is expressed by steroid cell tumors and is helpful in excluding other sex cord-stromal tumors [7].

Usually these tumors are benign, but 25-43% of steroid cell tumors are clinically malignant, with 20% of cases found to exhibit metastasis outside of the ovary [1, 4, 6]. Metastatic lesions usually occur within peritoneal cavity and rarely occur at distant sites [2]. A study by Hayes and Scully reported five pathological features predictive of malignancy: two or more mitosis per 10 high-power fields, necrosis, size of the tumor (more than 7 cm), hemorrhage, and grade 2 or 3 nuclear atypia [3].

In this case, the lesion was a finding during the study of infertility and none of the pathological features predictive of malignancy were present. If this tumor was the cause of infertility in this woman is not established, since there are reported cases of diagnosis in pregnant women and in our case the woman maintained regular menses [4, 6]. Also, there were already described cases of spontaneous pregnancies after removal of the tumor, not verified so far in this case [5, 8].

Steroid cell tumors NOS must be distinguished from other ovarian tumors and other steroid cell tumor types, in which proliferation of steroid hormone-producing cells occurs as a secondary event. It includes stromal luteoma, Leydig cell tumor, luteinized thecomas, pregnancy luteomas, and carcinomas [6]. Steroid cell tumors NOS are differentiated from Leydig cell tumors because of their deficiency of cytoplasmic Reinke crystals. Also, Leydig cell tumor is usually present in hilar location and it is commonly associated with Leydig cell hyperplasia. Stromal luteoma is confined to the ovarian stroma and frequently occurs in association with stromal hyperthecosis [9]. The steroid cell tumors NOS might have a fibromatous component like that of thecoma, but that component accounts for less than 10% of the tumor [10]. Pregnancy luteomas are more commonly multifocal, bilateral in one-third of cases, are usually discovered in patients at the time of cesarean section, and usually regresses after pregnancy [9].

The mainstay treatment of ovarian steroid cell tumors is total abdominal hysterectomy with bilateral salpingo-oophorectomy and complete surgical staging. But, for those who desire to preserve fertility, conservative surgery with unilateral oophorectomy can be accepted [2, 8]. As in the case described, the tumor was an unexpected finding, and a laparoscopic cystectomy was held with close surveillance of hormonal levels and imaging in the postoperative period. Another case of laparoscopic cystectomy in steroid cell tumors was already described by Jiang et al. and reported success in 3 years of follow-up without evidence of recurrence of the tumor [1]. The authors believe that performing a cystectomy allows the preservation of the ovarian reserve, important in a nullipara, and probably it is a good approach in these circumscribed tumors.

Patients with malignant tumors should undergo chemotherapy and radiotherapy after surgery, but given the rarity of this type of tumor, which is usually benign and rarely metastasizes or recurs, the optimal adjuvant regimen remains unknown. Gonadotropin releasing hormone agonist has also been considered because of its suppressive effect on ovarian steroidogenesis [1, 2, 4].

Regular follow-up evaluation with measurement of serum testosterone level is mandatory, but since little is known about the behavior of these tumors, it is not determined for how long [2, 4].

A uniqueness of this case, not yet described in the literature, was the performance of ovarian stimulation in the context of infertility treatment. No tumor recurrence was observed. On the other hand, despite the high doses of gonadotrophin, there was no follicular response of the ovary, despite the normal appearance of the remaining ovary and the contralateral ovary, probably due to other factors of the woman.
